# Health care needs, experiences, and satisfaction after terrorism: a longitudinal study of parents of survivors of the Utøya attack

**DOI:** 10.1186/s12913-024-10592-1

**Published:** 2024-03-08

**Authors:** Ida Frugård Strøm, Tore Wentzel-Larsen, Synne Stensland, Grete Dyb, Lise Eilin Stene

**Affiliations:** 1 Norwegian Centre for Violence and Traumatic Stress Studies, Pb 181 Nydalen, 0409 Oslo, Norway; 2 Centre for Child and Adolescent Mental Health, Eastern and Southern Norway, Oslo, Norway; 3 Research and Communication Unit for Musculoskeletal Health (FORMI), Division of Clinical Neuroscience, Oslo University Hospital, Oslo, Norway; 4 Institute of Clinical Medicine, University of Oslo, Oslo, Norway

**Keywords:** Post-terror health care needs, Unmet health care needs, Terror, Parents of survivors, Depression and anxiety, Posttraumatic stress, Social support, Physical health problems, Psychological reactions

## Abstract

**Background:**

There is scarce knowledge on the health care follow-up of parents of terror attack survivors. This study focused on the mothers and fathers of survivors and examined (1) their perceived health care needs relative to their psychological reactions, physical health problems (unmet health care needs), and adaptation to work; (2) whether sociodemographic characteristics, health problems and social support were associated with unmet health care needs; and (3) how unmet health care needs, sociodemographic characteristics, and experiences with health services associated with overall dissatisfaction during the health care follow-up.

**Methods:**

Interview and questionnaire data from three waves of the Utøya parent study were analyzed (*n* = 364). Chi-square tests and t- tests were used to compare unmet physical and psychological health care needs, sociodemographic factors and post-terror attack health reported by mothers and fathers. Logistic regression analyses were used to examine whether sociodemographic characteristics, unmet health care needs, and health care experiences were associated with overall dissatisfaction among mothers and fathers of the survivors during the health care follow-up.

**Results:**

Among the mothers, 43% reported unmet health care needs for psychological reactions, while 25% reported unmet health care needs for physical problems. Among the fathers, 36% reported unmet health care needs for psychological reactions, and 15% reported unmet health care needs for physical problems. Approximately 1 in 5 mothers and 1 in 10 fathers reported “very high/high” needs for adaptation to work. Poorer self-perceived health, higher levels of posttraumatic stress and anxiety/depression symptoms, and lower levels of social support were significantly associated with reported unmet psychological and physical health care needs in both mothers and fathers. Parents with unmet health care needs reported significantly lower satisfaction with the help services received compared to parents whose health care needs were met. Low accessibility of help services and not having enough time to talk and interact with health care practitioners were associated with overall dissatisfaction with the help received.

**Conclusions:**

Our findings highlight that parents of terror-exposed adolescents are at risk of having unmet psychological and physical health care needs and thus need to be included in proactive outreach and health care follow-up programs in the aftermath of a terror attack.

## Background

Acute stress reactions often recover spontaneously following trauma [1]. However, for some individuals, receiving appropriate health care to treat current conditions and to prevent the development of chronic disease can be crucial for their recovery. Experiencing a terror attack may have a potential long-lasting impact on survivors’ mental and somatic health, as well as their functioning [2–7]. Emerging evidence suggests that a significant number of survivors report unmet health care needs following exposure to a terror attack [8–10], which may persist years after the terror attack occurred [11–13]. The literature has primarily focused on survivors of terror attacks and focuses less on parents of survivors. Research conducted on parents of military personnel and ill and traumatized children, however, shows that parents also suffer from high levels of posttraumatic stress disorder (PTSD) symptoms and depression [14–18]. Still, there is scarce knowledge about parents’ experiences and satisfaction with the health care they receive. The successful recovery of young survivors depends on having well-functioning parents who can address and meet their children’s needs [19, 20]. Knowledge about potential unmet health care needs among parents of terror attack survivors, associated characteristics, and parents’ overall satisfaction with the help received may contribute to improving postdisaster health care follow-up for both survivors and their parents. As such, public health preparedness for future catastrophes should be strengthened. This study sought to examine these factors in parents of young survivors following the Utøya terror.

Following the Utøya terror attack, a national family-based outreach program was established during the first year, which was anchored in the existing health services of the municipalities. As survivors and their parents resided in different parts of Norway, the Norwegian Directorate of Health advised affected municipalities regarding how to organize and facilitate health care services and support for survivors and parents. The outreach program was based on the following three principles: (1) proactivity in early outreach, (2) continuity of follow-up, and (3) targeted interventions for individuals in need. It was recommended that all survivors and their families be appointed a contact person for one year, who was assigned to contact the affected family, offer personal meetings, assess the survivor’s functional level and help needs, and provide information about available help services and in specialist health care services in the municipality [18, 21].

Research after the Utøya attack demonstrated higher levels of both anxiety/depression and posttraumatic stress symptoms among parents of survivors compared to the general population [20]. This was partly due to the distress and guilt that the parents felt regarding their children’s traumatic experiences [20]. Studies following other traumatic events have also shown a high correlation between parental and child PTSD [22, 23]. Although we know little about parents’ health care needs following a terror attack, other findings from the Utøya study show that most of the parents (73%) were contacted through the national outreach program. However, the outreach program was less successful in reaching nontraditional families (e.g., “blended families”, “single-parent households”) and parents of non-Norwegian origin [18]. Furthermore, in line with research on terror attack survivors [8, 9, 24], parents with posttraumatic stress reactions in the early aftermath of the terror attack had a significantly higher frequency of general practitioner visits [25]. In addition, an increase in primary health care service consumption was observed for both mothers and fathers in the early aftermath following the terror attack, which persisted for 6–36 months after the attack [26]. An increase in specialized mental health care service utilization was also observed for mothers [26].

Given this information, more knowledge is needed about parents’ subjective experiences of post-terror attack health care and their overall satisfaction with the help received. Furthermore, characteristics that may be associated with potential unmet health care needs among parents need to be explored. Although there is no common consensus on the definition of unmet health care needs, it can be understood as the perceived need for health care but not receiving care because of potential barriers (e.g., financial concerns, lack of time, stigma) or not receiving the expected care [27, 28].

A model that may be useful in understanding these potential barriers is the Behavioral Model of Health Services Use (BM) [29], which comprises both individual and contextual determinants of health service use. The model includes three major components: predisposing factors (e.g., age, sex, education level, employment status, ethnicity, and marital status), enabling factors (e.g., finances to pay for health services, means of transportation to access health services and waiting times), and need factors (e.g., perceived and evaluated need for care). Health care may vary according to these components. For example, research has demonstrated that women experience more mental health consequences after trauma than men [30]. Moreover, women are generally more likely to utilize health care services than men [31, 32]. Furthermore, studies utilizing the BM in the US have found that individuals in ethnic minority groups are less likely to receive health care than white non-Hispanic individuals and that persons with lower education levels have less access to health care than persons with higher education levels [31]. Findings on finances and marital status, however, have been mixed. While some studies have found that unmarried women and financially disadvantaged individuals have less access to health care and lower rates of health care utilization, other studies have found that these same groups are more likely to receive care [31].

Drawing from the literature on the unmet health care needs of terror attack survivors, barriers to health care include general barriers, such as financial concerns, a lack of time and stigma, and postdisaster concerns, such as feeling that others are more in need of help than oneself or being too depressed or overwhelmed to seek help [8]. In addition, survivors with diagnoses and high symptom levels, low levels of social support and lower education and income levels are more likely to report having unmet health care needs [9, 13]. Social support from friends and family following a traumatic experience is a major protective factor against subsequent mental health problems [33–37] and unmet health care needs [9]. Research is lacking as to whether these same characteristics of survivors with unmet health care needs apply to parents of terror attack survivors.

To fill these knowledge gaps and provide insight that will contribute to improving postdisaster health care, the current study sought to examine aim (1) mothers’ and fathers’ perceived health care needs relative to psychological reactions and physical health problems (unmet health care needs) and their perceived needs for help and adaptation to work, aim (2) whether sociodemographic characteristics (age, ethnicity, financial status, education level, marital status and employment status), health problems, and social support are associated with unmet health care needs among mothers and fathers, and aim (3) how unmet health care needs, sociodemographic characteristics, and experiences with health services are associated with overall satisfaction with health care follow-up among mothers and fathers of terror attack survivors.

## Methods

### Study design and setting

Survivors were sent postal invitations with information about the study 4–5 months after the event (T1) and were subsequently contacted and asked to provide contact information for their parents. The parents of 482 survivors, aged 13–33 years at the time of the attack, were then contacted and asked if they were willing to participate in the study. Of these parents, 453 participated at T1. The parents participated by completing questionnaires or face-to-face interviews with trained health personnel. Parents of survivors born in 1992 or later were invited to participate in face-to-face interviews, while parents of survivors born before 1992 were invited to complete pen-and-pencil questionnaire due to restricted interviewer capacity. The same parents who were asked to participate at T1 were asked to participate in a follow-up study 14–15 months following the event (T2), of whom 426 participated (response rate: 94.0%). Parents who had participated at either T1 or T2 (*n* = 531) were subsequently asked to participate in the study three years after the terror attack (T3). In addition, three parents were self-recruited for the study at T3, resulting in a total of 534 parents. This study reported on parents who participated at T3, as questions about health care needs, experiences, and satisfaction with health care services were only asked at T3. Written informed consent was obtained from all participants. The study was approved by the Regional Committee for Medical and Health Research Ethics in Norway (for more details about the procedures, see [18]). All methods were carried out in accordance with the relevant guidelines and regulations in the Declaration of Helsinki.

### Participants

Of the 534 parents who participated in at least one of the three waves, 364 participated at T3 which constitute a response rate of 68.2%. Of these parents, the majority participated at both T1 and T2 (76%, *n* = 278), 13.1% (*n* = 48) participated only at T2, 10.1% (*n* = 37) participated only at T1 and 0.8% (*n* = 3) participated only at T3. In two cases, the child survivor had two female caregivers participating in the study. Further examination revealed that these cases involved a stepmother and a sister (in addition to the biological mother). Because we examined parental participation and we could only include one mother and father for each survivor, we included the biological mother in these two cases. The final sample comprised 223 mothers and 141 fathers who participated at T3 (*n* = 364). Of these parents, 98% were biological parents, while 2% were stepparents. Moreover, at T3, most of the survivors lived in a household comprising two biological parents (62%), while 20% lived with one biological parent and one stepparent, and 18% lived with one biological parent.

### Measures

Questions about health care needs, experiences, satisfaction with health care services and social support were only asked at T3. Measures of anxiety, depression and posttraumatic stress reactions were included at all time points (T1-T3). For sociodemographic characteristics, measures at T1 were prioritized (for more details, see below).

### Health care needs, experiences, and satisfaction with health care services (T3)

The questions about health care needs, experiences and satisfaction with health care services were developed and adapted from the National Survey of Patient Experiences in Norway designed by the Norwegian Knowledge Centre for Health Services [38]. The same measures were included in the survey for the survivors and have been published in a previous study [10].

### Health care needs relative to the help received (T3)

Respondents were asked whether they had felt that they needed help for their psychological reactions (no missing) or physical health problems (2 missing) following the Utøya attack and whether they had received help for these psychological reactions/physical health problems. They were also asked if they needed help to adapt to work or school (5 missing) and whether they had received such help. As the mean age of the parents was 48 years, it is likely that this measure largely related to their work and not their education; therefore, the term “adaptation to work” is used throughout the manuscript. The response options for all questions comprised “Not at all”, “To a small extent”, “To some extent”, “To a large extent”, and “To a very large extent”.

### Unmet health care needs for psychological reactions and physical health problems

The study sought to measure unmet health care needs in terms of not receiving sufficient care. Thus, if the parents’ perceived help needs were higher relative to the help that they received, they were registered as having unmet health care needs for psychological reactions (no missing) and physical health problems (4 missing), e.g., if a person reported having help needs to “some extent” and reported a lower value for received help, e.g., “to a small extent”, the person was registered as having unmet health care needs.

For *health care experiences*, we sought to measure the parents’ experiences with the health care received in relation to the health care follow-up after the Utøya attack by asking the following questions: *Did you find the help services to be well organized?* (14 missing). *Were you offered help without having to ask for it?* (6 missing). *How pleased are you with the accessibility of the help services?* (12 missing). *Were you treated with care and consideration?* (14 missing). *Were you involved in decisions regarding your treatment/follow-up?* (14 missing). *Did you have enough time to talk and interact with health care practitioners?* (18 missing). Response options included “Not at all”, “To a small extent”, “To some extent”, “To a large extent”, and “To a very large extent”. The measures were dichotomized to examine negative experiences in relation to overall dissatisfaction in the logistic regressions wherein “Not at all” and “To a small extent” were coded as 1 and the remaining response categories were coded as 0.

For *satisfaction*, we sought to measure parents’ overall satisfaction with their health care experiences in relation to the terror attack with the following question: *Overall, were the help and treatment you received after the terrorist attack satisfactory?* Response options included “Not at all”, “To a small extent”, “To some extent”, “To a large extent”, and “To a very large extent”. The measure was dichotomized to examine overall dissatisfaction in relation to different experiences with help services in the logistic regression, in which “Not at all” and “To a small extent” were coded as 1 and the remaining response categories were coded as 0 (11 missing).

### Other health-related measures (T3)

*Self-perceived health* was measured by asking the respondents, “*Overall, do you consider your health to be* “Excellent”, “Very good”, “Good”, “Somewhat good” or “Not good?” (1 missing).

*Current health compared to health before the attack* was measured with the following question: *Compared to before the terror attack, how would you say your overall health is now?* Response options included “Much better”, “A little better”, “Similar”, “A little worse”, and “Much worse” (1 missing).

*Had to wait to be offered help* was measured by asking the respondent, “*Did you have to wait to be offered help from public services after the terrorist attack?*” Response options were “No”, “Yes, but not for long”, “Yes, quite long”, “Yes, far too long”, and “Not offered help” (8 missing).

*Perceived benefit of help* was measured by asking the question, “*Overall, what benefit have you had from the help you received from public services after the terrorist attack?*” The response options included “No benefit”, “Little”, “Some”, “Much”, “Very much”, and “Did not get help” (7 missing).

### Psychological assessments (T1-T3)

*Posttraumatic stress symptoms (PTSS)* were measured with a customized (in collaboration with the authors of the index) UCLA PTSD Symptom Index for Children and Adolescents [39] to cover the 20 diagnostic criteria in the fifth edition of the Diagnostic and Statistical Manual of Mental Disorders (DSM-5 [40]): reexperience (five items), avoidance (two items), negative alterations in cognition and mood (seven items), and arousal and reactivity (six items). Participants were asked to rate how much they had been bothered by each symptom over the past month using a 5-point Likert scale ranging from 0 (“never”) to 4 (“most of the time”). Mean scores for PTSS were computed for T1 (Cronbach’s alpha, α: 0.91), T2 (α: 0.93) and T3 (α: 0.93) (for more details see Stensland et al. [41]) (missing T1:52, T2:40,T3:no missing).

*Anxiety and depression symptoms* were measured using the Hopkins Symptom Checklist-8 (HSCL-8), which is a short version of the HSCL-25 that has been shown to have high psychometric qualities in population-based studies [10, 42]. The HSCL-8 measures symptoms of depression/anxiety in the past 14 days using eight items, scored from 1 (not bothered) to 4 (very bothered) [43]. Mean scores were calculated for T1 (α: 0.90), T2 (α: 0.92) and T3 (α: 0.93) missing T1:52, T2:41, T3:no missing).

*Perceived social support* (FSSQ: [44]) was only measured at T3 and comprised receiving attention, care and support from loved ones; being cared for when sick; receiving advice and support from others regarding school, work or personal matters; and being included in social activities with others. The respondents were asked to rate each statement on a 5-point Likert scale as follows: “*As much as I would like*” (5); “*Almost as much as I would like*” (4); “*Somewhat, but would like more*” (3); “*Less than I would like*” (2); and “*Much less than I would like*” (1). Mean scores were calculated [36]. Cronbach’s alpha was 0.89 (3 missing).

### Sociodemographic characteristics (T1)

Sociodemographic variables were based on T1 data. However, for participants who had not participated at T1, answers at T2 (*n* = 48) or T3 (*n* = 3) were used to reduce missing data. Sociodemographic characteristics comprised gender (no missing), age at the time of the attack (1 missing), ethnicity (no missing), financial situation (no missing), education level (no missing), living situation (no missing), and employment/education status (no missing). *Non-Norwegian origin* was defined as being born abroad. *Financially disadvantaged* was defined as having a financial status “below the average” (compared to average or above). *Higher education* was defined as having completed up to 4 years or more than 4 years of university or college. *Living with a spouse or partner* (category 1) was compared to living without a spouse or partner, with or without other adults or children (category 0). *Not working or in school* was measured at all waves and was defined as reporting not being at work and in school during at least one of the waves (T1-T3).

### Statistical methods

**Fig. 1:**
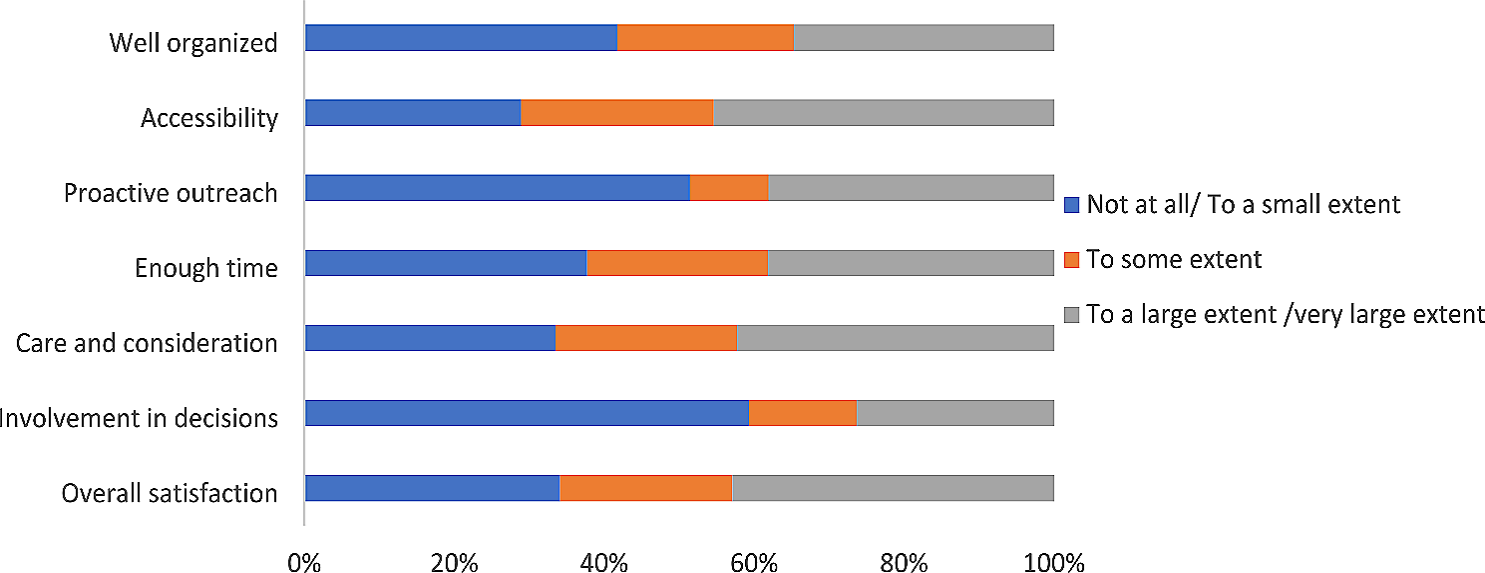
Mothers’ experiences and satisfaction with the post-terror attack help services received (*n* = 223)

**Fig. 2:**
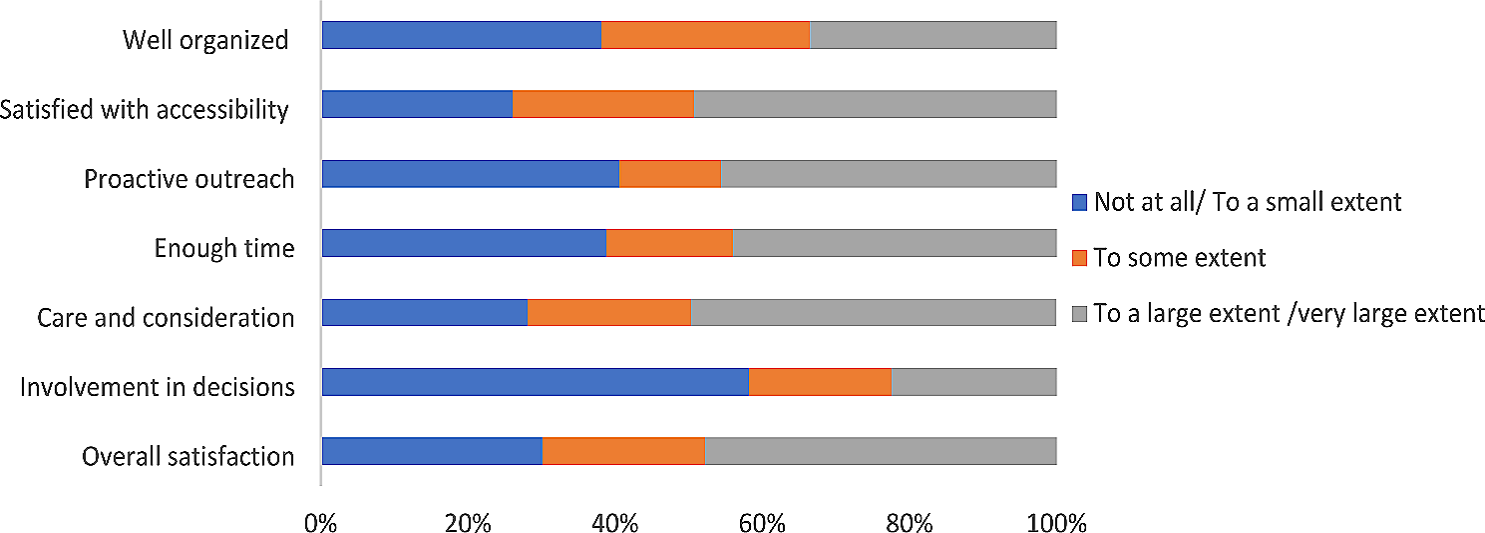
Fathers’ experiences and satisfaction with the post-terror attack help services received (*n* = 141)

The sociodemographic characteristics and post-terror attack health-related variables of the mothers and fathers were compared (Tables 1 and 2). All other analyses were run separately for mothers and fathers. Chi-square tests and t-tests were used to examine and compare (1) mothers’ and fathers’ perceived health care needs relative to their psychological reactions, physical health problems, and adaptation to work (aim 1, Table 2), (2) characteristics associated with mothers’ and fathers’ unmet health care needs (aim 2, Tables 3 and 4), and (3) mothers’ and fathers’ experiences with health care services (Figs. 1 and 2). Cohens’ d and Cramer’s V were also included for characteristics associated with mothers’ and fathers’ unmet health care needs (Tables 3 and 4) to measure and report effect sizes. Unadjusted logistic regression analyses were used to examine whether sociodemographic characteristics, unmet health care needs, and the various health care experiences were associated with overall satisfaction with the health care follow-up among the mothers and fathers of the terror attack survivors (aim 3, Tables 5). The following benchmarks for interpreting the effect sizes were used: about 1.5 to 1 = small effect (or weak association), about 2.5 to 1 = medium (or moderate), about 4 to 1 = large (or strong), about 10 to 1 = very large (or very strong) [45]. The logistic regression analyses used multiply imputed data with 200 imputations based on the dependent and independent variables in the analyses, separately for mothers and fathers, Attrition analyses were previously conducted [26]. The results indicated that fathers were more likely to drop out than mothers at T3 than during previous waves. No significant differences were found for non-Norwegian origin, levels of early posttraumatic stress and the dropout rate [26]. IBM SPSS Statistics 27, Stata 16 and R were used for all analyses. Multiple imputation used the R package mice.

**Table 1:** Sociodemographic characteristics of the study sample (*N* = 364)

Sociodemographic Characteristic	Total (*N* = 364) % (n)	Fathers (*n* = 141)	Mothers (*n* = 223)	*p* value
Age at the time of the attack, mean (SD)	47.9 (6.0)	49.7 (5.9)	46.8 (5.7)	< 0.001
Non-Norwegian origin	8.0 (29)	7.8 (11)	8.1 (18)	0.672
Financially disadvantaged	14.3 (52)	11.3 (16)	16.1 (36)	0.473
Higher education level	58.2 (212)	56.0 (79)	59.6 (133)	0.465
Living with a spouse or partner	80.5 (293)	86.5 (122)	76.7 (171)	0.361
Not working or in school during at least one of the study waves	14.6 (53)	11.3 (16)	16.6 (37)	0.630

Tests: There were 114 families with data for both the mother and father, 109 families with data for the mother only and 27 families with data for the father only. For the continuous variable “age”, the test was based on a linear mixed effects model (function lme in R package nlme). For the other variables, tests were based on a generalized mixed effects model for dichotomous variables (function glmmTMB with family binomial in R package glmmTMB)

**Table 2:** Characteristics of the study sample (*N* = 364)

Characteristics of parents of survivors	Total (*N* = 364) % (n)	Fathers (*n* = 141)	Mothers (*n* = 223)	*p* value
**Health Post-Terror Attack**				
**Self-perceived health (T3)**:				0.018
Excellent	11.8 (43)	12.8 (18)	11.3 (25)	
Very good	40.5 (147)	46.8 (66)	36.5 (81)	
Good	28.9 (105)	28.4 (40)	29.3 (65)	
Somewhat good	13.2 (48)	7.8 (11)	16.7 (37)	
Not good	5.5 (20)	4.3 (6)	6.3 (14)	
**Current health compared to health before the attack (T3)**:				0.033
Much better	2.2 (8)	2.1 (3)	2.3(5)	
A little better	6.3 (23)	7.8 (11)	5.4 (12)	
Similar	58.7 (213)	63.8 (90)	55.4 (123)	
A little worse	26.7 (97)	22.0 (31)	29.7 (66)	
Much worse	6.1 (22)	4.3 (6)	7.2 (16)	
**Perceived health care needs for psychological reactions**:				< 0.001
Very high	8.2 (30)	3.5 (5)	11.2(25)	
High	15.1 (55)	12.8 (18)	16.6 (37)	
Some	28.3 (103)	14.9 (21)	36.8 (82)	
Low	19.8 (72)	27.7 (39)	14.8 (33)	
No	28.6 (104)	41.1 (58)	20.6(46)	
**Perceived health care needs for physical health problems**:				< 0.001
Very high	3.6 (13)	2.9 (4)	4.1 (9)	
High	9.1 (33)	2.9 (4)	13.1 (29)	
Some	16.0 (58)	11.4 (16)	18.9 (42)	
Low	10.8 (39)	7.9 (11)	12.6 (28)	
No	60.5 (219)	75.0 (105)	51.4 (114)	
**Perceived needs for adaptation to work or school**:				0.003
Very high	5.8 (21)	4.3 (6)	6.8 (15)	
High	10.3 (37)	7.9 (11)	11.9 (26)	
Some	18.9 (68)	13.6 (19)	22.4 (49)	
Low	12.8 (46)	13.6 (19)	12.3 (27)	
No	52.1 (187)	60.7 (85)	46.6 (102)	
**Had to wait to be offered help**:				.201^a^
No	48.3 (172)	52.9 (73)	45.5 (99)	
Yes, but not for long	18.8 (67)	17.4 (24)	19.7 (43)	
Yes, for quite long	5.6 (20)	4.3 (6)	6.4 (14)	
Yes, for too long	5.1 (18)	3.6 (5)	6.0 (13)	
Not offered help	22.2 (79)	21.7 (30)	22.5 (49)	
**Perceived benefit of help**:				
No benefit	6.4 (23)	5.0 (7)	7.4 (16)	.416^a^
Little	17.1(61)	17.9 (25)	16.6 (36)	
Some	26.9 (96)	25.7 (36)	27.6 (60)	
Much	22.7 (81)	24.3 (34)	21.7 (47)	
Very much	11.8 (42)	9.3 (13)	13.4 (29)	
Did not get help	15.1 (54)	17.9 (25)	13.4 (29)	
**Overall satisfaction**:				0.245
Very high	11.9 (42)	11.0 (15)	12.4 (27)	
High	32.9 (116)	36.8 (50)	30.4 (66)	
Moderate	22.7 (80)	22.1 (30)	23.0 (50)	
Small	15.3 (54)	14.7 (20)	15.7 (40)	
No	17.3 (61)	15.4 (21)	18.4 (217)	

Tests: There were 114 families with data for both the mother and the father, 109 families with data for the mother only and 27 families with data for the father only. Tests were likelihood ratio tests for cumulative link mixed models (function clmm2 in R package ordinal)

^a^ Warning of a possible convergence issue

**Table 3:** Characteristics associated with mothers’ unmet health care needs at T3 (*n* = 223)

Characteristic	Perceived needs higher than the help received													
	Psychological reactions (*n* = 223)							Physical problems (*n* = 222)						
	Unmet health care needs, *n* = 96 %, Mean (n/SD)	Health care needs met *n* = 127 %, Mean (n/SD)	*P* value		Cohens’ d		Cramer’s V	Unmet health care needs, *n* = 56 %, Mean (n/SD)	Health care needs met, *n* = 166 %, Mean (n/SD)	*P* value	Cohens’ d	Cramer V		
***Sociodemographic Characteristic***														
Age at the time of the attack	46.9 (5.51)	46.8 (5.9)	0.888		0.019			47.2 (5.22)	46.7 (5.9)	0.530	0.91			
Non-Norwegian origin	11.5 (11)	5.5 (7)	0.106				0.108	5.4 (3)	9.0 (15)	0.383		0.059		
Financially disadvantaged	17.7 (17)	15.0 (19)	0.581				0.037	17.9 (10)	15.7 (26)	0.700		0.026		
Higher education level	52.1 (50)	65.4 (83)	**0.045**				0.134	58.9 (33)	59.6 (99)	0.925		0.006		
Living with a spouse or partner	74.0 (71)	78.7 (100)	0.403				0.056	73.2 (41)	78.3 (130)	0.433		0.053		
Not working full-or part-time	15.6 (15)	17.3 (22)	0.736				0.023	10.7 (6)	18.7 (31)	0.167		0.093		
***Health Post-Terror Attack***														
**Self-perceived health (T3)**:														
Excellent/very good	39.6 (38)	54.0 (68)	.**025**				0.183	39.3 (22)	50.3 (83)	0.254		0.111		
Good	29.2 (28)	29.4 (37)						37.5 (21)	26.7 (44)					
Somewhat good/not good	31.3 (30)	16.7 (21)						23.2 (13)	23.0 (38)					
**Current health compared to health before the attack (T3)**:														
Poorer	47.9 (46)	28.6 (36)	.**011**				0.202	53.6 (30)	31.5 (52)	**0.013**		0.199		
Same	46.9 (53)	61.9 (78)						41.1 (23)	60.0 (99)					
Better	5.2 (5)	9.5 (12)						5.4 (3)	8.5 (14)					
**Mean posttraumatic stress symptoms score**:														
Wave 1	1.41 (0.63)	1.16 (0.69)	**0.010**		0.374			1.54 (0.60)	1.18 (0.68)	**< 0.001**	0.542			
Wave 2	1.22 (0.60)	0.96 (0.73)	**0.006**		0.384			1.29 (0.62)	0.99 (0.70)	**0.005**	0.441			
Wave 3	1.15 (0.67)	0.79 (0.68)	**< 0.001**		0.536			1.17 (0.64)	0.87 (0.70)	**0.003**	0.443			
**Mean anxiety/depression score**:														
Wave 1	1.96 (0.58)	1.82 (0.68)	0.129		0.215			2.11 (0.60)	1.81 (0.64)	.**004**	0.483			
Wave 2	1.80 (0.66)	1.63 (0.68)	0.078		0.251			1.86 (0.64)	1.64 (0.68)	**0.036**	0.335			
Wave 3	1.79 (0.65)	1.50 (0.65)	**< 0.001**		0.450			1.80 (0.62)	1.57 (0.67)	**0.022**	0.348			
**Social support**:														
Wave 3	4.0 (0.90)	4.47 (0.63)	**< 0.001**		-584			3.86(0.93)	4.42 (0.69)	**< 0.001**	.-742			

**Table 4:** Factors associated with fathers’ potential unmet health care needs (*n* = 141)

Characteristics	Perceived needs higher than the help received									
	Psychological reactions (*n* = 141)					Physical problems (*n* = 138)				
	Unmet health care needs, *n* = 50 %, Mean (n/SD)	Health care needs met, *n* = 91 %, Mean (n/SD)	*P* value	Cohens’ d	Cramer’s V	Unmet health care needs, *n* = 22 %, Mean (n/SD)	Health care needs met, *n* = 116 %, Mean (n/SD)	*P* value	Cohens’ d	Cramer’s V
***Sociodemographic Background***										
Age at the time of the attack	48.59 (5.13)	50.26 (6.20)	0.091	-286		50.7 (5.25)	49.50 (6.06)	0.355	0.198	
Non-Norwegian origin	12.0 (6)	5.5 (5)	0.168		0.116	13.6 (2)	6.9 (8)	0.285		0.091
Financially disadvantaged	16.0 (8)	8.8 (8)	0.197		0.109	9.1 (2)	12.1(14)	0.689		0.034
Higher education level	64.0 (32)	51.6 (47)	0.157		0.119	77.3 (17)	53.4 (62)	**0.038**		0.176
Living with a spouse or partner	82.0 (41)	89.0 (81)	0.243		0.098	86.4 (19)	86.2 (100)	0.984		0.002
Not working full-or part-time	12.0 (6)	11.0 (10)	0.856		0.015	27.3 (6)	8.6 (10)	0.012		0.213
***Health Post-Terror Attack***										
**Self-perceived health (T3)**:										
Excellent/very good	42.0 (21)	69.2 (63)	**0.003**		0.291	36.4 (8)	63.8 (74)	**< 0.001**		0.326
Good	36.0 (18)	24.2 (22)				27.3 (6)	28.4(33)			
Somewhat good/not good	22.0 (11)	6.6 (6)				36.4 (8)	7.8 (9)			
**Current health compared to health before the attack (T3)**:										
Poorer	32.0 (16)	23.1 (21)	0.357		0.121	50.0 (11)	21.6 (25)	**0.019**		0.240
Same	56.0 (28)	68.1 (62)				45.5 (10)	67.2 (78)			
Better	12.0 (6)	8.8 (8)				4.5 (1)	11.2 (13)			
**Mean posttraumatic stress symptoms score**:										
Wave 1	1.16 (0.62)	0.66 (0.46)	**< 0.001**	0.979		1.36 (0.59)	0.72 (0.50)	**< 0.001**	1.246	
Wave 2	1.18 (0.74)	0.59 (0.48)	**< 0.001**	1.004		1.47 (0.61)	0.66 (0.58)	**< 0.001**	1.377	
Wave 3	1.08 (0.68)	0.50 (0.48)	**< 0.001**	1.037		1.25 (0.66)	0.59 (0.56)	**< 0.001**	1.148	
**Mean anxiety/depression score**:										
Wave 1	1.74 (0.57)	1.35 (0.38)	**< 0.001**	0.866		1.88 (0.50)	1.41 (0.45)	**< 0.001**	1.032	
Wave 2	1.81 (0.71)	1.32 (0.40)	**< 0.001**	0.912		2.02 (0.67)	1.39 (0.50)	**< 0.001**	1.179	
Wave 3	1.70 (0.62)	1.25 (0.35)	**< 0.001**	0.968		1.87 (0.68)	1.33 (0.43)	**< 0.001**	1.156	
**Social support**:										
Wave 3	3.84 (0.89)	4.53 (0.51)	**< 0.001**	.-1.021		3.69 (0.81)	4.40 (0.68)	**< 0.001**	-1.018	

## Results

### Characteristics of the study sample

The mothers and fathers had similar sociodemographic characteristics, except for age. The mothers were younger (by 3 years on average) at the time of the attack (Table 1).

Mothers reported higher perceived health care needs for psychological reactions and physical health problems in the two-and-a-half-year period following the Utøya attack than fathers (Table 2). More specifically, 25 (11.2%) mothers reported having “very high” health care needs for psychological reactions, i.e., three times as often as fathers. For physical health problems, 17.2% of the mothers reported having “very high” or “high” health care needs compared to 5.8% of the fathers. Some parents also had a hard time adapting to work after the terror attack; 19% of the mothers and 12% of the fathers reported that they had “very high” or “high” needs for adaptation to work after the terror attack.

Although most of the parents perceived their health as good or very good at T3, a minority reported that it was not good or somewhat good (23% of mothers, 12% of fathers). However, when looking at current health compared to before the attack, approximately one-third of the parents reported that their current health was worse than their health before the terror attack. Furthermore, while the majority of the parents reported that they did not have to wait to be offered help or that they did not wait for long, approximately 1 in 5 mothers and fathers reported that they had not been offered help. Furthermore, approximately 1 in 5 mothers and fathers reported that the help received had no or little benefit.

### Parents’ health care needs relative to the physical and psychological health care received

Among the mothers, 43% (*n* = 96) reported higher perceived health care needs for psychological reactions relative to the help that they received (unmet health care needs), while 25% (*n* = 56) reported unmet health care needs for physical problems (Table 3).

Among the fathers, 36% (*n* = 50) reported unmet health care needs for psychological reactions, and 16% (*n* = 22) reported unmet health care needs for physical problems (Table 4).

### Characteristics of parents’ perceived unmet health care needs

Sociodemographic characteristics did not significantly differ between mothers with perceived unmet and met health care needs for psychological reactions and physical problems, except for level of education (Table 3). Mothers with perceived unmet health care needs for psychological reactions reported a lower prevalence of having higher education compared to mothers whose health care needs were met.

However, mothers who reported unmet health care needs reported poorer health after the terror attack (at T3) compared to before the attack, higher levels of posttraumatic stress symptoms at all timepoints, and anxiety and depression symptoms and lower levels of social support at T3 compared to mothers whose health care needs were met. This was similar for both psychological and physical problems. In addition, for unmet health care needs for psychological reactions, mothers also reported having poorer self-perceived health. Effect sizes for continuous variables were in the medium range, except for age where it was small. For categorical variables effect sizes were small or medium.

There were no significant differences in sociodemographic characteristics between fathers reporting unmet and met health care needs, except for level of education, similar to the mothers. Fathers with perceived unmet health care needs for physical problems, reported lower prevalence of having higher education compared to fathers whose health care needs were met (Table 4). Fathers with perceived unmet health care needs reported having poorer self-perceived health, higher levels of posttraumatic stress, anxiety, and depression symptoms at all timepoints, and lower levels of social support at T3 compared to fathers who felt that their psychological and physical health care needs were met. Fathers who reported unmet health care needs for physical problems also reported poorer current health compared to before the attack. Effect sizes for continuous variables were medium or large, except for age where it was small for physical problems. For categorical variables effect sizes were small or medium.

### Parents’ experiences with help services after the Utøya attack

While many parents were satisfied with the help received, approximately 1 in 3 mothers and fathers reported little or no satisfaction with the help services received after the Utøya attack (see Fig. 1). The mothers reported, to a smaller extent, being involved in decisions, receiving proactive outreach, and feeling that the help services were not well organized. The fathers reported, to a smaller extent, being involved in decisions, receiving proactive outreach, and having enough time to talk and interact with health care practitioners (see Fig. 2).

### Parents’ satisfaction with help services after the Utøya attack

To explore the relationship between the different experiences of the help services received after the attack, sociodemographic characteristics, unmet health care needs and overall dissatisfaction for parents, logistic regression analyses were conducted. For both mothers and fathers, sociodemographic characteristics were not significantly associated with overall dissatisfaction (Table 5). Also, there were small effect sizes, ranging from 0.5 to 1.95 which indicate weak associations. Mothers with unmet health care needs for psychological reactions and fathers with unmet health care needs for both physical and psychological problems had significantly higher odds of overall dissatisfaction with the help services received compared to parents whose health care needs were met. The effect size for mothers were strong, while the effects size for fathers were moderate. Answering “not at all” or “to a small extent” on questions on different experiences with the health services assessed in the study was associated with higher odds of overall dissatisfaction with the help services received post-terror attack. Also, there were very large effect sizes. For example, mothers who reported not receiving proactive outreach had 27 times higher odds of overall dissatisfaction compared to mothers who received proactive outreach.

**Table 5:** Logistic regression models for associations between sociodemographic characteristics, unmet health care needs, experiences with health care services and overall dissatisfaction with help services, using multiply imputed data

	Mothers (*n* = 223) Unadjusted		Fathers (*n* = 141) Unadjusted	
	OR (95% CI)	*p* value	OR (95% CI)	*p* value
**Sociodemographic Characteristic**				
Age (per 5 years) at the time of the attack	0.95 (0.75–1.22)	0.693	1.04 (0.76–1.42)	0.801
Non-Norwegian origin	0.64 (0.21–1.99)	0.440	1.93 (0.55–6.80)	0.301
Financially disadvantaged	1.95 (0.93–4.10)	0.077	0.70 (0.21–2.35)	0.566
Higher education level	0.59 (0.34–1.05)	0.073	0.85 (0.41–1.77)	0.665
Living with a spouse or partner	0.70 (0.37–1.35)	0.291	0.51 (0.18–1.42)	0.193
Not working full-or part-time	1.48 (0.71–3.08)	0.290	1.37 (0.46–4.07)	0.574
**Unmet health care needs**				
Psychological reactions	5.21 (2.83–9.57)	< 0.001	3.85 (1.78–8.30)	< 0.001
Physical problems	1.78 (0.94–3.36)	0.076	3.96 (1.52–10.34)	0.005
**Experiences with health care Services**				
Not well organized	14.32 (7.12–28.80)	< 0.001	21.75 (8.44–56.06)	< 0.001
Not pleased with accessibility	24.17 (10.96–53.31)	< 0.001	47.20 (15.04–14.81)	< 0.001
Not enough time	26.53 (12.42–56.70)	< 0.001	116.25 (24.72-546.63)	< 0.001
No proactive outreach	27.88 (11.18–69.56)	< 0.001	14.63 (5.83–36.71)	< 0.001
No care and consideration	16.39 (8.10-33.15)	< 0.001	28.37 (10.31–78.08)	< 0.001
No involvement in decisions	6.91 (3.35–14.27)	< 0.001	11.21 (3.69–34.03)	< 0.001

Based on 200 imputed data sets for all variables in the regression analyses, separately for mothers and fathers

*Note*. Response options for experiences with health care services were “Not at all/to a small extent vs. to some/to a large/to a very large extent”. Outcome variable, missing *n* = 5 (fathers) and 6 (mothers). Experiences with help services: missing ranging from 0 to 4

## Discussion

This study adds new knowledge about the unmet health care needs of parents of terror attack survivors and their satisfaction with the health care received after a terror attack. While many parents had their health care needs met, a significant minority of parents reported unmet psychological and physical health care needs following the terror attack. More than 1 in 3 mothers and fathers reported unmet health care needs for psychological reactions, while almost 1 in 4 mothers and 1 in 7 fathers reported unmet health care needs for physical problems. These findings suggest that several parents did not receive sufficient care in the aftermath of the terror attack. This is of concern given that receiving appropriate care may be crucial for successful recovery in both parents *and* survivors, as the recovery of young survivors may depend on having well-functioning parents who can address and meet their needs [19, 20]. These findings also emphasize the importance of considering both physical and psychological health problems in the follow-up of parents after their children experience a traumatic event, even when their child is an adolescent or a young adult, as in this study.

The frequency of unmet health care needs among parents in this study was higher than that among the survivors of the Utøya terror attack [10] and survivors of other terror attacks [9]. However, different from this study, the latter study defined unmet health care needs as “not receiving needed mental health care or counseling during the last 12 months”, of which 4.2% of the study participants (*n* = 36,625) answered affirmatively. Thus, the latter study had a shorter study period compared to the current study, comprising survivors and a much larger cohort of terror-exposed individuals. The study of the Utøya survivors, found that 1 in 5 survivors reported unmet psychological health care needs, while 1 in 7 survivors reported unmet health care needs for their physical health problems [10]. The high prevalence of perceived unmet health care needs among parents demonstrates that there is a potential for improvement of the postdisaster health care follow-up of parents. However, it is important to note that we could not determine whether the unmet health care needs measured in this study were due to not receiving sufficient care or whether they were due to dissatisfaction with the help received.

To better understand unmet health care needs among parents, we sought to examine associated characteristics as well as overall satisfaction with the help received. Our results revealed that some parents were more likely to perceive their need for help as greater than the help received (unmet health care needs). As described above, mothers reported unmet health care needs more often than fathers. This may be explained by women reporting more symptoms in general [46]. A prior study demonstrated that mothers indeed had higher utilization of mental health services after the Utøya terror attack than fathers [26]. This may indicate that the higher levels of perceived unmet needs among mothers than fathers may have been due to higher needs for care rather than receiving less care [26].

Mothers and fathers without higher education had higher frequency of reporting unmet health care needs. This aligns with studies using the BM model, finding that individuals with lower education levels have less access to health care than persons with higher education levels [31].

Furthermore, poorer self-perceived health, higher levels of posttraumatic stress and anxiety/depression symptoms, and lower levels of social support were significantly associated with reporting unmet psychological and physical health care needs among both mothers and fathers. This is similar to findings of health care needs in survivors [9, 13] and underscores the need for targeted interventions, including among parents.

Nevertheless, it is important to note from previous research that having a prior mental health diagnosis may be associated with both subjective unmet mental health care needs and a greater use of mental health services [9]. Previous studies of the Utøya attack showed that parents with posttraumatic stress reactions in the early aftermath of the terror attack had a significantly higher frequency of general practitioner visits, as well as an increase in primary health care service consumption and an increase in specialized mental health care service consumption [25, 26]. Thus, it is important to highlight that this study captured the parents’ subjective unmet health care needs and did not include objective measure of the actual help received. Many parents also reported excellent or very good self-perceived health despite having unmet health care needs, which underscores that they may have been coping well despite having perceived unmet health care needs. It also suggests that some parents had moderate unmet health care needs.

Social support following a traumatic experience is one of the most important protective factors for recovery [33–37]. Brackbill et al. [9] underscored that the link between low levels of social support and unmet health care needs may mediate the long-term health effects in survivor’s post-trauma. Our study further highlights that parents with low levels of social support reported more unmet health care needs, emphasizing the importance of social support in the aftermath of a terror attack.

In addition to health care needs, the findings from this study underscore that some parents needed help to adapt to work in the aftermath of the terror attack and beyond. Almost 1 in 5 mothers reported having a high or very high need for adaptation to work following the terror attack. Along with the finding of social support being associated with fewer unmet health care needs, this suggests that a more comprehensive follow-up, beyond addressing health care needs, is needed.

Finally, while many parents were satisfied with the help received, approximately 1 in 3 mothers and fathers reported little or no satisfaction with the help received. To gain a better understanding of this, we examined whether sociodemographic characteristics, unmet health care needs, and different aspects of the services received could explain the parents’ levels of dissatisfaction. While sociodemographic characteristics were not significantly associated with overall satisfaction with help services, parents with unmet health care needs reported significantly higher overall dissatisfaction with the help services received after the Utøya attack compared to parents whose health care needs were met. In addition to testing for significance, effect sized were considered. The effect size was particularly strong for mothers with unmet health care needs for psychological reactions, whom had over five times higher odds of being dissatisfied with the help services provided compared to mothers whose health care needs were met. This indicates that the unmet health care needs measured in this study relate not only to internal factors, such as psychological health problems but also to the quality or quantity of the help services provided. The corresponding associations for fathers were of moderate effect size.

As expected, parents who answered “not at all” or “to a small extent” for questions regarding their different experiences with health services were also more likely to be dissatisfied with the help services overall, as confirmed by the very strong effect sizes. Aspects of the help services related both to the structural aspects of the services (such as how well the service was organized and how accessible it was), as well as the quality of the service (such as having enough time to talk and interact with health care practitioners, receiving proactive outreach, providers showing care and consideration, and being involved in decisions).

For prevention purposes and to strengthen public health preparedness for future catastrophes, it is important to examine which aspects of the services for parents were related to their overall dissatisfaction. Mothers who reported, to a smaller extent, receiving proactive outreach and being given enough time to talk and interact with health care practitioners, and being pleased with accessibility to the services were more likely to be dissatisfied with the help services overall. Fathers who reported, to a smaller extent, being given enough time to talk and interact with health care practitioners, being pleased with the accessibility of the services, and providers showing care and consideration, had the highest odds of overall dissatisfaction. This points to important targeted improvements for future catastrophes and subsequent follow-up to provide appropriate care to treat current conditions and to prevent the development of chronic disease in survivors and their parents.

### Limitations and future directions

A limitation of the study is that we were unable to determine the timing of the health services usage. Parents were asked about their health care needs, experiences, and satisfaction with health care services over a period of more than two years. Thus, we could not differentiate their evaluation of the services received in the immediate aftermath and the care received years after the attack. The long observation period may also result in recall bias. The subjective measures of unmet health care needs limit our results, as they may not be a true reflection of the objective help received. Thus, future studies should include objective measures of health care usage. Furthermore, there is no consensus on how to measure unmet health care needs and satisfaction, as it may involve multiple factors, such as expectations of care, preferences for care and the quality of care received. Having these measures prior to the attack could help to better determine and differentiate the health care received pre- and post-terror attack. The types of physical and psychological health problems were not specified and should be explored in future studies. Furthermore, we lacked information about the divorce rate among parents following the attack and could not assess whether this was associated with their unmet health care needs. Previous research from the Utøya study showed that a higher number of individuals of non-Norwegian origin were lost to follow-up [10]. This suggests a selection bias and that non-Norwegian parents may be less represented. Language barriers may also have impacted non-Norwegian parents’ access to health care and should be examined in future studies. Moreover, information from the parents of the youngest survivors was collected through interviews by health personnel, while parents of the eldest survivors answered questionnaires. The different participation modes might have introduced bias in the likelihood of reporting positive or negative health care experiences. The external validity of this study is limited, as Norway offers universal health care. This contrasts with countries with privatized health care, which depends on the personal economy to a larger extent. More research is needed to explore differences and similarities between various health care systems and subsequent post-terror attack follow-up.

Ideally, adjusted analyses should have been conducted to examine the associations among unmet health care needs, sociodemographic characteristics, and overall satisfaction. However, this was not possible due to the limited sample size. Future studies should include information about surviving children’s characteristics, such as their psychological health (e.g., PTSR), somatic health and physical injuries, to examine how such characteristics may be associated with their parents’ access to health care and evaluations of the health care received. A previous study utilizing Utøya data, however, found that early posttraumatic stress reactions in children were not significantly associated with an overall increase in primary physician visits among parents (for more information, please see Haga et al. [25]).

Despite these limitations, the study also has strengths. All the survivors of the Utøya attack were identified by the police. As such, we had a defined study population and were able to contact most of the survivors’ parents. This differs from other terror attack studies where many survivors were unknown [10]. Parents were invited to participate in the study regardless of the health care received, while previous studies often only included recipients of specific services [10, 47]. However, to gain knowledge about how to improve postdisaster health care follow-up, it is important to also include individuals who did not receive care [10].

## Conclusions

Our findings highlight that parents of young terror attack survivors are at risk of having unmet psychological and physical health care needs. Some parents may also need help to adapt to work. Poorer self-perceived health, higher levels of posttraumatic stress and anxiety/depression symptoms, and lower levels of social support were significantly associated with reporting unmet psychological and physical health care needs among both mothers and fathers. For mothers, not having a higher level of education was also significantly associated with having unmet psychological health care needs, while for fathers it associated with having unmet physical health care needs. Parents with unmet health care needs also reported significantly lower satisfaction with the help services received after the Utøya attack compared to parents whose health care needs were met. This specifically related to accessibility, time spent with providers, and proactive outreach. This points to important targeted areas of improvement for future health care follow-up for parents of terror attack survivors. Our findings indicate that interventions following psychological trauma should not only address the mental health, but also the physical health and the potential need for adaptations at work. The findings further highlight the relevance of assessing parents’ social support, their level of education, and their satisfaction with the follow-up, as having low social support, lower levels of education, and being dissatisfied with the help services may increase the risk of unmet healthcare needs. As for theory development, the study suggests that future research should examine to what extent unmet needs were due to lack of help or perceived poor quality of the help received. Finally, more information should be developed about the type of physical health needs and work adaptions needed among parents of youth exposed to severe trauma.

## Data Availability

The datasets generated and/or analyzed during the current study are not publicly available due to privacy and ethical restrictions but are available from the corresponding author upon reasonable request.

## Abbreviations

BM: Behavioral Model of Health Services Use
DSM-5: Diagnostic and Statistical Manual of Mental Disorders
FSSQ: Functional Social Support Questionnaire
HSCL-8: Hopkins Symptom Checklist-8
PTSD: Posttraumatic stress disorder
PTSS: Posttraumatic stress symptoms
T: Time
UCLA: University of California, Los Angeles
